# Identifying Antioxidant Proteins by Combining Multiple Methods

**DOI:** 10.3389/fbioe.2020.00858

**Published:** 2020-07-23

**Authors:** Xianhai Li, Qiang Tang, Hua Tang, Wei Chen

**Affiliations:** ^1^School of Pharmacy, Chengdu University of Traditional Chinese Medicine, Chengdu, China; ^2^Innovative Institute of Chinese Medicine and Pharmacy, Chengdu University of Traditional Chinese Medicine, Chengdu, China; ^3^School of Life Sciences, Center for Genomics and Computational Biology, North China University of Science and Technology, Tangshan, China

**Keywords:** antioxidant, reduced amino acid composition, g-gap dipeptide composition, feature selection, support vector machine

## Abstract

Antioxidant proteins play important roles in preventing free radical oxidation from damaging cells and DNA. They have become ideal candidates of disease prevention and treatment. Therefore, it is urgent to identify antioxidants from natural compounds. Since experimental methods are still cost ineffective, a series of computational methods have been proposed to identify antioxidant proteins. However, the performance of the current methods are still not satisfactory. In this study, a support vector machine based method, called Vote9, was proposed to identify antioxidants, in which the sequences were encoded by using the features generated from 9 optimal individual models. Results from jackknife test demonstrated that Vote9 is comparable with the best one of the existing predictors for this task. We hope that Vote9 will become a useful tool or at least can play a complementary role to the existing methods for identifying antioxidants.

## Introduction

Reactive oxygen species (ROS) are composed of oxygen free radicals and nitrogen free radicals. Free radicals contain unpaired electron molecules or atoms, which are generally unstable and highly reactive. They can trigger lipid peroxidation during metabolism, which leads to DNA strand breaks, and even oxidize biofilms and almost all molecules in tissues indiscriminately. Fortunately, organisms have evolved effective strategies to detect and prevent molecular oxygen metabolites (Finkel and Holbrook, 2000; Mccord, 2000; Klaus and Heribert, 2004; Li et al., 2015). This is called the antioxidant system of organisms, which can effectively resist the damages caused by ROS (Agus et al., 2011).

Owing to their important roles in the antioxidant system, natural antioxidants have received more and more attentions (Yigit et al., 2014). Antioxidant proteins can neutralize free radicals, thereby blocking cell damage or death caused by free radicals. The consumption of antioxidants can be used to reduce the oxidative stress caused by excessive ROS, and reduce the damage to the organism (Yang et al., 2017). Antioxidants have also been applied to prevent diseases such as heart disease, cancer, cardiovascular disease (Gey, 1990; Dreher and Junod, 1996; Diaz et al., 1997). Its unique role in anti-aging was also reported (Ames et al., 1993).

Accordingly, many proteins extracted from rapeseed, ginkgo and other plant seeds are used as natural antioxidants (Nichole et al., 2008; Huang et al., 2009). Some micronutrients such as vitamin C and vitamin E (Lobo et al., 2010) are also considered as antioxidant molecules. However, our body cannot synthesize these nutrients, so we need to ingest them from the diet. Therefore, it has become an urgent task to identify proteins with antioxidant activity from natural compounds.

Although identifying antioxidant proteins through biochemical experiments is an objective and accurate method, they are still labor intensive and expensive. With the massive production of protein sequences, a series of computational methods have been proposed to identify antioxidant proteins. For the first time, Enrique et al. (2013) proposed a random forest model for predicting antioxidant proteins based on star map topological index and achieved satisfactory results. However, their model was trained based on a dataset including redundant sequences that might lead to overestimation problems (Chou, 2011). In 2013, Feng et al. (2013) constructed a high quality dataset with the sequence similarity less than 60%. Based on this dataset, they developed a Naive Bayes method by using the optimal dipeptides and obtained an average accuracy of 66.88%. Based on this dataset, a series of methods have been proposed in recent years. In 2016, Feng et al. (2016) proposed a support vector machine based method, called AodPred, which identifies antioxidant by using the optimal 3-gap dipeptide features and improves the prediction accuracy to 74.79%. Later on, Lei et al. (2018) developed a computational model called SeqSVM by using support vector machine and obtained an overall accuracy of 89.46%. More recently, Meng et al. (2019) proposed another support vector machine model called AOPs-SVM by integrating multiple kinds of features and obtained an overall accuracy of 94.2%. However, the sensitivity of AOPs-SVM is only 68%.

The above results indicate that the prediction accuracy still needs to be improved. Therefore, in this study, based on the optimal dipeptide composition and the reduced amino acid composition (Chen D. et al., 2012; Chen W. et al., 2012; Feng et al., 2016; Lv et al., 2019), a new model was constructed. The results show that the performance of the proposed method for identifying antioxidant proteins is better than or at least comparable to existing methods.

## Materials and Methods

### Training Set and Test Set

The dataset used in the present work is the same as the one used by Feng et al. (2013, 2017),which includes 253 antioxidant protein sequences and 1552 non-antioxidant protein sequences with the sequence identity less than 60%. The dataset is expressed as:

(1) S=S∪+S-

where “S” stands for benchmark dataset, “S_+_” is the positive dataset and contains 253 antioxidant protein sequences, and “S_–_” is the negative dataset and contains 1552 non-antioxidant protein sequences. The longest and shortest peptides in the dataset are 1463 and 11 amino acids, respectively.

In the following analysis, the dataset S was divided into two parts. One of them is the training set S_T_ and includes 80% of the sequences in S, and the remaining 20% sequences form the testing set S_E_, which are expressed as following,

(2) S=TS0.8+*∪S0.8-*

(3) S=ES-ST

### Independent Dataset

To objectively evaluate the proposed method and compare with its counterpart, an independent dataset was built in the present work. By searching the Universal Protein Resource (Uniprot) with the keywords “antioxidant” and “reviewed,” and setting the date from March 1, 2014 to March 31, 2020, we obtained 22 antioxidant protein sequences that are independent from the sequences in the dataset S.

### Support Vector Machine

Support Vector Machine (SVM) is a method for effectively identifying data according to supervised learning method, which is widely used in bioinformatics and other fields (Feng et al., 2016; Liao et al., 2018; Wang et al., 2019; Liu and Chen, 2020). If the samples are linearly separated, the basic idea of the SVM algorithm is to solve the separation hyperplane that can correctly divide the training dataset and have the largest geometric interval; when the samples are nonlinearly separated, SVM maps the low-dimensional data to the high-dimensional data by the kernel function space. In this work, the LIBSVM package downloaded from https://www.csie.ntu.edu.tw/~cjlin/libsvm/ was used to perform the prediction. The best regularization parameter *C* and kernel width parameter *g* were determined by using the grid search method.

### Sequence Representation

#### g-gap Dipeptide Composition

The *g*-gap dipeptide composition was proposed to describe the long-range correlation between two amino acid residues and has been proved to be effective in the field of protein recognition (Ding et al., 2013; Lin et al., 2013; Tan et al., 2019). Accordingly, in the present work, the *g*-gap dipeptide composition was used to encode the sequences in both benchmark dataset and independent test dataset.

The g-gap dipeptide composition is expressed as following,

(4) $$F={\left[f_{1}^{g}\,f_{2}^{g}\,\ldots \,f_{i}^{g}\,\ldots \,f_{400}^{g}\right]}^{T}$$

(5) $$f_{i}^{g}=\frac{n_{i}^{g}}{L-g-1}$$

where $f_{\text{i}}^{\text{g}}$ represents the frequency of the *i*-th (*i* = 1, 2,…, 400) dipeptide with *g*-gap interval in the protein sequence, and T represents the transposition of the vector. $n_{\text{i}}^{\text{g}}$ represents the number of the *i*-th *g*-gap dipeptide. In the present work, *g* is an integer in the range of [0, 9]. For example, *g* = 0 represents the correlation between two adjacent amino acid residues, and *g* = 1 represents the correlation of two amino acid residues separated by one residue, and so forth.

#### Reduced Amino Acid Composition

With the aim of including structural information, the reduced amino acid composition (RAAC) was applied to encode proteins (Feng et al., 2016). Compared with the classical amino acid composition, the RAACs can reduce protein complexity and eliminate part of the redundant signals without losing sequence information intact (Wang and Wang, 1999; Liu et al., 2018). In order to obtain the RAAC from the sequences, Zuo et al. (2017) established the online webserver and database (Zheng et al., 2019) that can be used to calculate RAAC.

In term of RAAC, based on amino acid sequence and structure information, the 20 natural amino acids can be aggregated into a smaller number of representative amino acid residues (Thomas and Dill, 1996; Mirny and Shakhnovich, 1999; Solis and Rackovsky, 2000). According to the different optimization procedures (Op) for protein sequences proposed by Etchebest et al. (2007), there are 5 different cluster files for the 20 natural amino acids, i.e., Op(5), Op(8), Op(9), Op(11)and Op(13), which are formulated as below:

Op⁢(i)=

(6) $$\left\{ \begin{array}{c} \text{Op}\,\left(5\right):\left\{G;\mathrm{IVFYW};\mathrm{ALMEQRK};P;\mathrm{NDHSTC}\right\}\\ \text{Op}\,\left(8\right):\left\{G;\mathrm{IV};\mathrm{FYW};\mathrm{ALM};\mathrm{EQRK};P;\mathrm{ND};\mathrm{HSTC}\right\}\\ \text{Op}\,\left(9\right):\left\{G;\mathrm{IV};\mathrm{FYW};\mathrm{ALM};\mathrm{EQRK};P;\mathrm{ND};\mathrm{HS};\mathrm{TC}\right\}\\ \text{Op}\left(11\right):\left\{G;\mathrm{IV};\mathrm{FYW};A;\mathrm{LM};\mathrm{EQRK};P;\mathrm{ND};\mathrm{HS};\right\}\\ T;C\}\\ \text{Op}\left(13\right):\{G;\mathrm{IV};\mathrm{FYW};A;L;M;E;\mathrm{QRK};P;\mathrm{ND};\\ \mathrm{HS};T;C\} \end{array}\right.$$

where *i* indicates the different cluster profiles (*i* = 5, 8, 9, 11, 13), and the letters between the two semicolons belong to the same cluster.

Accordingly, a sequence can be encoded based on the reduced amino acid composition. As indicated in Eq. 6, for the *n*-peptide composition with various cluster profiles, the components and dimensions of the feature vector will be different.

(7) $$\Psi ={\left[\Psi_{1},\Psi_{2},\cdots ,\Psi_{\Omega }\right]}^{T}$$

where Ω is the dimension of the vector, and is based on the selected *n* and cluster profiles. For example, for the dipeptide composition with the cluster profile of Op(5), the Ω will be 25. In the current work, our initial tests demonstrate that the optimal *n* for different cluster profiles is as following,

(8) $$\Omega =\left\{ \begin{array}{c} 5^{3{}^{{}_{=}}}\,125\,f\,o\,r\,O\,p\,\left(5\right)\,c\,l\,u\,s\,t\,e\,r\\ 8^{2{}^{{}_{=}}}\,64\,f\,o\,r\,O\,p\,\left(8\right)\,c\,l\,u\,s\,t\,e\,r\\ 9^{2{}^{{}_{=}}}\,81\,f\,o\,r\,O\,p\,\left(9\right)\,c\,l\,u\,s\,t\,e\,r\\ 11^{2{}^{{}_{=}}}\,121\,f\,o\,r\,O\,p\,\left(11\right)\,c\,l\,u\,s\,t\,e\,r\\ 13^{2{}^{{}_{=}}}\,169\,f\,o\,r\,O\,p\,\left(13\right)\,c\,l\,u\,s\,t\,e\,r \end{array}\right.$$

### Performance Evaluation

There are usually three methods for evaluating the performance of computational models, namely independent dataset test, k-fold cross-validation test, and jackknife test (Wei et al., 2017; Chen et al., 2019; Manavalan et al., 2019a, b; Yang et al., 2019; Hasan et al., 2020; Lv et al., 2020). Among the three evaluation methods, the most rigorous and least random jackknife test was used to evaluate the proposed method.

The sensitivity (Sn), specificity (Sp), accuracy (Acc) and Mathew’s correlation coefficient (MCC) was selected as the evaluation metrics that are defined as following,

(9) $$S\,n=\frac{T\,P}{T\,P+F\,N}$$

(10) $$S\,p=\frac{T\,N}{T\,N+F\,P}$$

(11) $$A\,c\,c=\frac{T\,P+T\,N}{T\,P+F\,N+T\,N+F\,P}$$

(12) $$M\,C\,C=\frac{T\,N^{*}\,T\,P-F\,P^{*}\,F\,N}{\sqrt{{\left(T\,P+F\,P\right)}^{*}\,{\left(F\,N+T\,N\right)}^{*}\,{\left(T\,P+F\,N\right)}^{*}\,\left(T\,N+F\,P\right)}}$$

where TP, FP, FN, and TN represent true positive, false positive, false negative and true negative, respectively.

### Feature Selection

The principle of analysis of variance (ANOVA) is to measure the characteristic variance by calculating the ratio (*F*-value) between the characteristics of the groups and the internal characteristics of the groups (Lin and Ding, 2011; Basith et al., 2019). The larger the *F*-value, the greater the probability that each sample comes from a different population. In order to exclude redundant features and enhance the robustness of the proposed model, the ANOVA that widely used in computational proteomics (Ding et al., 2013; Lin et al., 2013; Basith et al., 2020) combined with the incremental feature selection (IFS) strategy was used to select the optimal features.

### Flowchart of the Method

**FIGURE 1:**
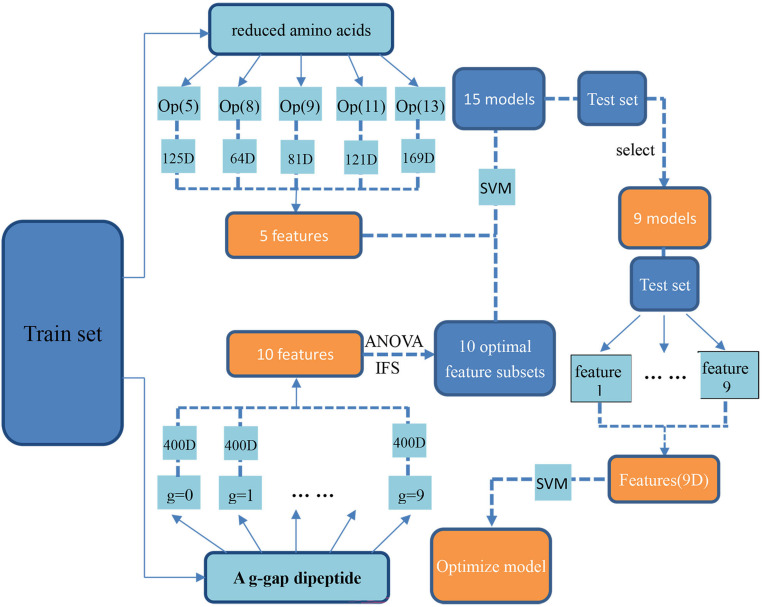
The flowchart of building the proposed method. The samples in the training dataset were firstly encoded by using reduced amino acid compositions and the optimal g-gap dipeptide compositions, respectively. Accordingly, 15 SVM models based on these different kinds of features was built. After validating the combinational performance of these models on the test dataset, 9 of the 15 SVM models were selected out as the optimal models. Finally, the SVM outs of these 9 models were used as the new features and used as the inputs of the SVM for building the proposed model.

By following the above procedure, we proposed a new computational method for identifying antioxidants. The flowchart of how to build it was shown in Figure 1.

## Results and Discussion

### Prediction Performance

**FIGURE 2:**
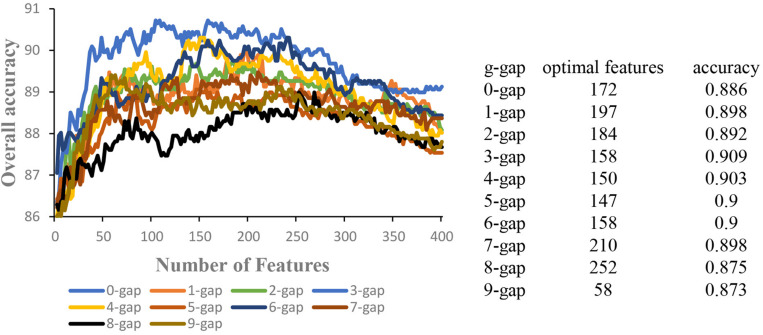
The IFS curves of different g-gap dipeptides (g = 0, 1, 2,…, 9). The optimal number of features and the accuracy based on the optimal features were shown in the right of the figure.

In order to obtain the optimal features, for a given kind of g-gap dipeptide composition, the 400 g-gap dipeptide compositions were ranked based on their F-scores. Each of the 400 dipeptide compositions were added one by one from higher to lower rank. This procedure was repeated 400 times, and for each time a SVM model was built. The accuracies of these models were then used to plot the IFS curve. Accordingly, the 10 IFS curves for *g* = 0 to 9 were obtained (Figure 2), where the abscissa is the number of features and the ordinate is the corresponding accuracy. In each curve, the optimal number of features were obtained when the curve reaches its peak. The optimal number of features and the accuracy based on the optimal features were shown in the right of Figure 2. Accordingly, 10 models were obtained based on g-gap dipeptide compositions.

**FIGURE 3:**
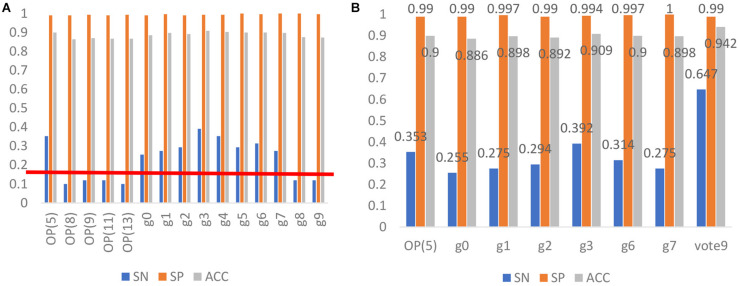
**(A)** The performance of the 15 models for identifying antioxidants. OP (5) stands for the method of optimizing amino acid residues to divide 20 amino acid letters into 5 categories, and then uses LIBSVM to establish a classification model. Using the method of g-gap dipeptide (Feng et al., 2016), we selected the best feature subset of protein sequence steps g = 0, 1… 7 to construct a g0, g1… g7 classification model. Vote9 is a comprehensive classification model that used the prediction results of the above classification models as feature vectors. **(B)** Comparison between Vote9 and single classification model.

Based on the reduced amino acid composition, another five models were built for identifying antioxidants. Their predictive performances together with that of the 10 models based on g-gap dipeptide composition were indicated in Figure 3A.

According to the prediction results of the 15 models, we removed 6 models with the sensitivity less than 20%. Therefore, 9 models were left and were combined to build the final model in the following analysis. To do so, the out of the nine SVM based models (1 or −1) were further used as the input of the SVM. Therefore, each sequence will be re-encoded by a 9-dimension vector with the element of 1 or −1. The model thus obtained is called Vote9. In the jackknife test, Vote9 obtained an accuracy of 0.94 with the sensitivity of 0.65, specificity of 0.99 and MCC of 0.74.

### Comparison With Single Model

In order to demonstrate the better performance of Vote9, we compared its performance with that of the single model for identifying antioxidants in the test dataset. The result is shown in Figure 3B. It was found that the sensitivity, specificity and accuracy of Vote9 are all significantly better than those of any single model, demonstrating that it’s necessary to built the model by combining the optimal single models.

### Comparison With Existing Methods

**FIGURE 4:**
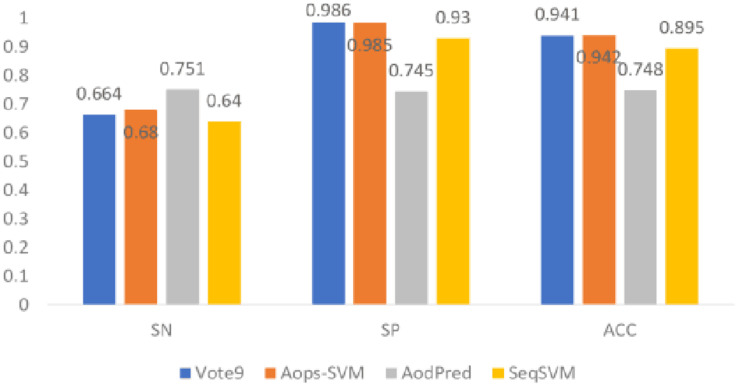
Comparison of Vote9 with existing methods.

In this section, we compared the performance of Vote9 with the performance of other existing methods (Aops-SVM, AodPred, and SeqSVM) that all trained based on the same dataset. Their performances were shown in Figure 4.

It was found that the accuracy of Vote9 is better than that of AodPred and SeqSVM, and is comparable with that of Aops-SVM. Although the sensitivity of Vote9 is lower than that of Aops-SVM and AodPred, its specificity is higher than that of the other three methods (Aops-SVM, AodPred, and SeqSVM). This result indicate that Vote9 might also become a useful tool for identifying antioxidants.

In order to objectively evaluate the performance of different methods for identifying antioxidants, a comparison was performed based on the independent dataset. Since some of the previous methods didn’t provide publicly available tool or doesn’t work properly, the comparison was also performed among Vote9, Aops-SVM, and AodPred. Their performances for identifying antioxidants in independent dataset were reported in Table 1. As shown in Table 1, we found that Aops-SVM performs the best, and Vote9 and AodPred can be used as complementary tools.

**TABLE 1:** Comparative results of different methods for identifying antioxidants in independent dataset.

**Sample**	**Aops-SVM**	**Aodpred**	**Vote9**	**Sample**	**Aops-SVM**	**Aodpred**	**Vote9**
P9WQB7	Y	Y	N	P9WIS6	Y	N	N
P9WHH9	Y	N	N	P9WQB6	Y	Y	N
P9WIS7	Y	N	**Y**	P9WID9	Y	Y	N
P9WG35	Y	Y	N	O17433	Y	Y	N
P9WGE9	Y	Y	N	P9WIE0	Y	N	N
P9WQB5	Y	Y	N	P9WID8	Y	Y	N
P9WIE3	Y	Y	N	P9WGE8	Y	Y	N
P0CU34	Y	Y	N	C0HK70	Y	Y	N
Q5ACV9	N	N	N	P9WQB4	Y	Y	N
P9WHH8	Y	N	**Y**	P9WG34	Y	Y	N
P9WIE1	Y	N	**Y**	P9WIE2	Y	Y	N

### Conclusion

The role of antioxidant proteins in neutralizing free radicals and preventing the damage of free radicals to cells is well known. Unfortunately, there are very few molecules with antioxidant properties in nature. Therefore, in order to accelerate researches on antioxidant proteins, there is an urgent need to develop effective methods for identifying them.

In the present work, we proposed a new method, called Vote9, in which the sequences were encoded by using the features generated from 9 optimal individual models. Results from jackknife test demonstrated that Vote9 is comparable with the best of the existing predictors for this task. The results of independent dataset test demonstrate that Vote9 can play a complementary role to the existing methods in this area. We hope that Vote9 will become a useful method for identifying antioxidants.

## Data Availability Statement

All datasets presented in this study are included in the article/supplementary material.

## Author Contributions

WC conceived and designed the experiments. XL, QT, and HT performed the experiments. XL and WC wrote the manuscript. All authors read and approved the final manuscript.

## Conflict of Interest

The authors declare that the research was conducted in the absence of any commercial or financial relationships that could be construed as a potential conflict of interest.
